# Integrated dataset on acute phase protein response in chicken challenged with *Escherichia coli* lipopolysaccharide endotoxin

**DOI:** 10.1016/j.dib.2018.09.103

**Published:** 2018-10-17

**Authors:** Anita Horvatić, Nicolas Guillemin, Haider Kaab, Dorothy McKeegan, Emily O’Reilly, Maureen Bain, Josipa Kuleš, Peter David Eckersall

**Affiliations:** a VetMedZg Laboratory, Faculty of Veterinary Medicine, University of Zagreb, Zagreb, Croatia; b Institute of Biodiversity, Animal Health & Comparative Medicine, College of Medicine, Veterinary Medicine and Life Sciences, University of Glasgow, Glasgow, UK

## Abstract

Data herein describe the quantitative changes in the plasma proteome in chickens challenged with lipopolysaccharide (LPS), a bacterial endotoxin known to stimulate the host innate immune system obtained by shotgun quantitative proteomic tandem mass tags approach using high-resolution Orbitrap technology. Statistical and bioinformatic analyses were performed to specify the effect of bacterial endotoxin. Plasma from chicken (N=6) challenged with *Escherichia coli* (LPS) (2 mg/kg body weight) was collected pre (0 h) and at 12, 24, 48, and 72 h post injection along with plasma from a control group (N=6) challenged with sterile saline. Protein identification and relative quantification were performed using Proteome Discoverer, and data were analysed using R. Gene Ontology terms were analysed by the Cytoscape application ClueGO based on *Gallus gallus* GO Biological Process database, and refined by REVIGO. Absolute quantification of several acute phase proteins, e.g. alpha-1-acid glycoprotein (AGP), serum amyloid A (SAA) and ovotrensferrin (OVT) was performed by immunoassays to validate the LC-MS results. The data contained within this article are directly related to our research article”*Quantitative proteomics using tandem mass tags in relation to the acute phase protein response in chicken challenged with Escherichia coli lipopolysaccharide endotoxin”* [1]. The raw mass spectrometric data generated in this study were deposited to the ProteomeXchange Consortium via the PRIDE partner repository with the dataset identifier PXD009399 (http://proteomecentral.proteomexchange.org/cgi/GetDataset?ID=PXD009399).

**Specifications table**

**Table:** 

Subject area	*Veterinary medicine, Biomedicine*
More specific subject area	*Proteomics, statistics, bioinformatics, immunoassays*
Type of data	*Excel files, graphs, figures*
How data was acquired	1. *LC-MS/MS analysis was performed using Ultimate 3000 RSLCnano system (Dionex, Germering, Germany) coupled to Q Exactive Plus mass spectrometer (Thermo Fisher Scientific, Bremen, Germany)*. 2. *Acute phase proteins absolute quantification was performed using ELISA tests (for AGP, SAA) and radial immunodiffusion (for OVT)*.
Data format	*Integration of raw and analyzed data*
Experimental factors	*Non-depleted plasma samples*
Experimental features	*Quantitative proteomic, bioinformatic and immunoassay analyses of chicken serum*
Data source location	*University of Glasgow Cochno Farm & Research Centre, Glasgow, United Kingdom*
Data accessibility	*The mass spectrometry proteomics raw data have been deposited to the ProteomeXchange Consortium via the PRIDE partner repository with the dataset identifier PXD009399MS* (http://proteomecentral.proteomexchange.org/cgi/GetDataset?ID=PXD009399). *All other data are available within this article.*

**Value of the data**

• This data provides information about changes in plasma proteome in chickens challenged with *Escherichia coli* lipopolysaccharide during 72 h with the emphasis on acute phase proteins such as alpha-1-acid glycoprotein (AGP), serum amyloid A (SAA) and ovotrensferrin (OVT).
• Peptide/protein information and pathway analysis datasets might be useful as a basis for future targeted analysis of proteins deregulated during inflammation.
• The data can be useful for other researchers investigating inflammation or pathophysiological mechanisms in veterinary medicine as well as in biomedical research.

## 1 Data

Protein and peptide identifications, as well as their corresponding peptide spectrum matches (PSMs), obtained by label-based proteomic approach, in plasma from chicken challenged with *Escherichia coli* lipopolysacharride (LPS) endotoxin (2 mg/kg body weight) pre (0 h) and at 12, 24, 48, and 72 h post injection along with plasma from a control group (N=6) challenged with sterile saline are reported, with the corresponding peptide spectrum matches (PSMs). Furthermore, relative quantification data after statistical analysis together with subsequent pathway analysis results and immunoassays data are also presented.

Results of analyze performed on this dataset has been represented in different figures and tables included in this Data in Brief article.

**Fig. 1:**
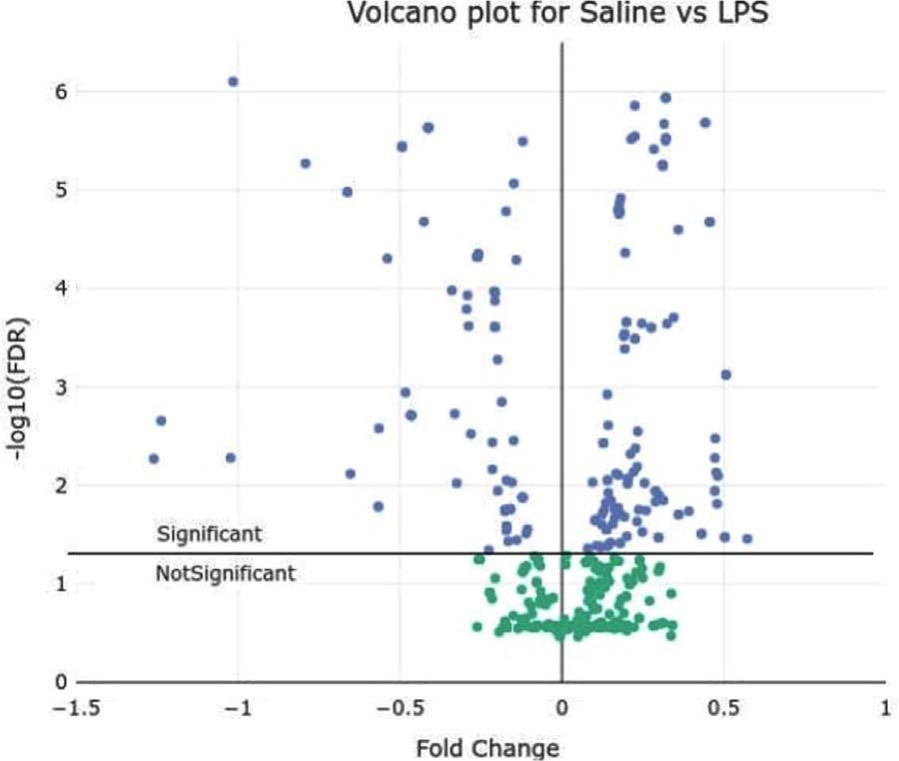
Volcano plot for chicken challenged with *Escherichia coli* lipopolysaccharide endotoxin *versus* saline group. Volcano plot of fold changes (x-axis) and their associated log10 transformed p-values (y-axis) for the 571 peptides analysed by LC-MS. Peptides significantly different between saline and LPS groups (log10 p>1.3) are in black, non-significant peptides (log10 p<1.3) are in grey.

Fig. 1 represent fold changes of proteins between LPS-treated and saline groups, and their associated p-values.

**Fig. 2:**
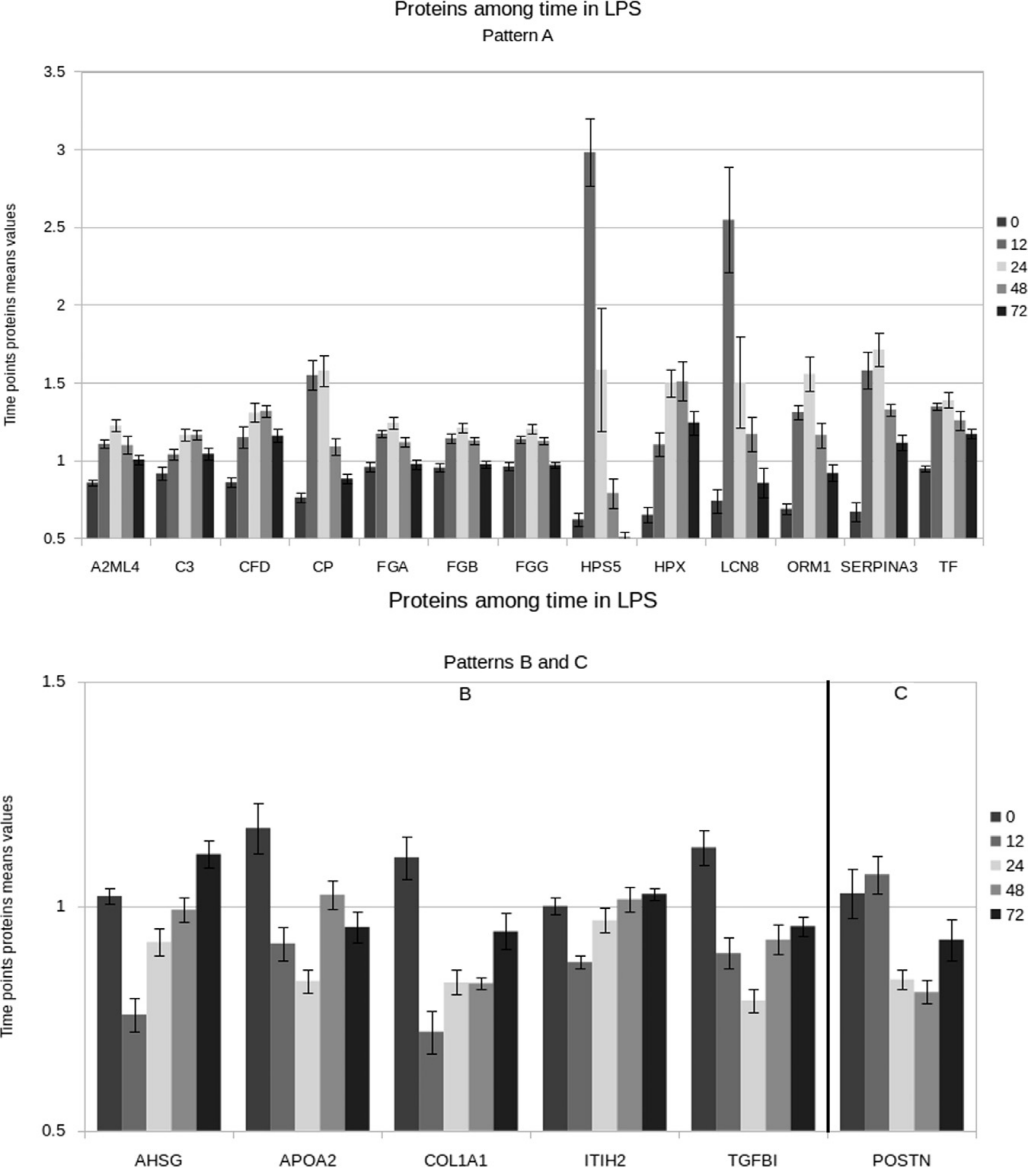
Time-affected proteins in chicken challenged with *Escherichia coli* lipopolysaccharide endotoxin (LPS group). Barplot of the mean and SEM of 19 proteins differentially expressed for the different time points (0 h, 12 h, 24 h, 48 h, 72 h) in LPS group. Proteins have been grouped according to their pattern of expression: A or B and C. Patterns have been defined according to the evolution of fold changes among time.

Fig. 2 represent how time affect proteins quantities in LPS animals.

**Fig. 3:**
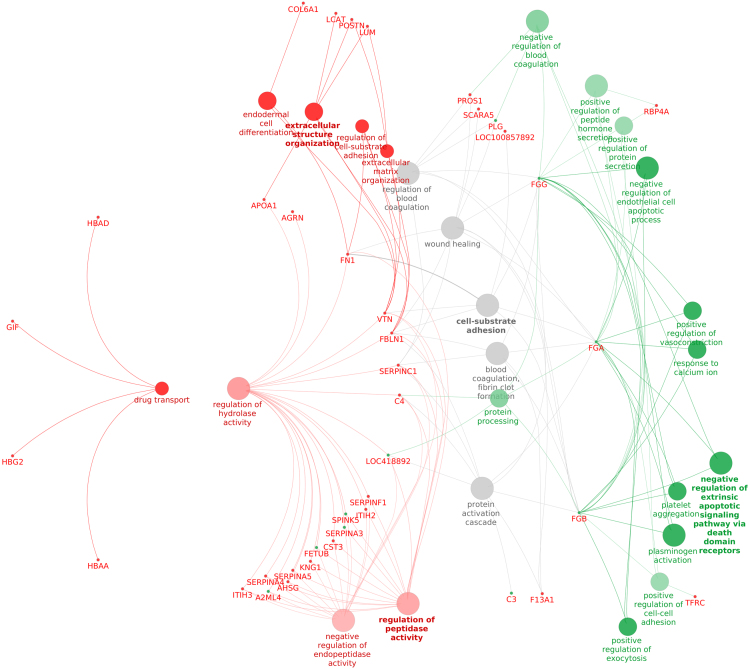
Interactome of pathways differentially expressed between chicken challenged with *Escherichia coli* lipopolysaccharide endotoxin and saline, and their intermediate proteins. Gene ontology analysed pathways and proteins over-represented in LPS compared with saline samples. This analyse have been done with the Cytoscape application ClueGO and the REVIGO tool for GO terms selection. GO terms and proteins over-expressed in LPS are in green, lower-expressed in LPS are in red. GO terms in grey could not be attributed specifically to over or lower expressed terms/proteins. GO terms in bold represent GO terms selected to be the most representative of their GO group defined by the REVIGO tool. The yFiles radial layout algorithm was applied.

Fig. 3 represent pathways up and down regulated, associated with LPS treatment.

**Fig. 4:**
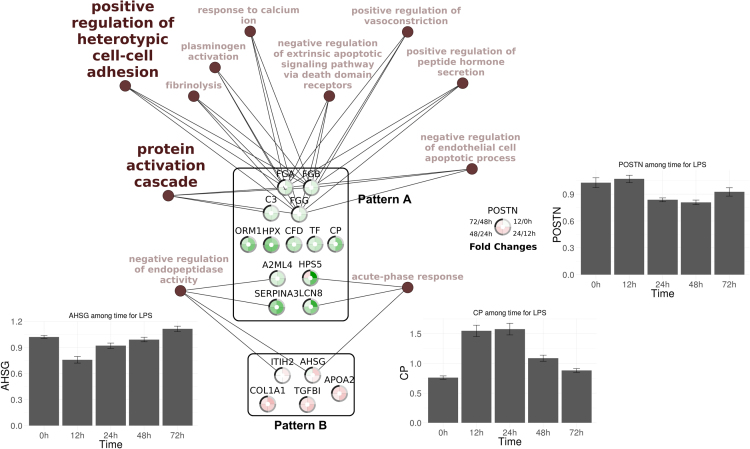
Interactome of pathways differentially expressed among time in chicken challenged with *Escherichia coli* lipopolysaccharide (LPS group) and their intermediate proteins. Gene ontology analysed pathways over-represented in the list of 19 proteins differentially expressed among time in LPS group. This analyse have been done with the Cytoscape application ClueGO and the REVIGO tool for GO terms selection. GO terms in bold represent GO terms selected to be the most representative of their GO group defined by the REVIGO tool. For each proteins, 4 fold changes among the 5 different time points have been represented using colour intensity to figure fold change value. Positive fold changes are in green, negative are in red, fold change values close to 0 are in white. Proteins have been gather in 3 groups defined by their fold changes pattern. The A pattern correspond to a quick increase of a protein, then go back to the initial situation, while the pattern B correspond to a quick decrease of a protein and then a go back to the initial situation. The C pattern correspond to a decrease which happen later in the infection process. For each pattern, evolution of one protein mean among time has been represented with histogram to illustrate the pattern properties.

Fig. 4 represent pathways affected by time, associated with LPS treatment. Evolution of proteins fold changes (LPS vs saline groups) are represented for each time-affected proteins.

**Fig. 5:**
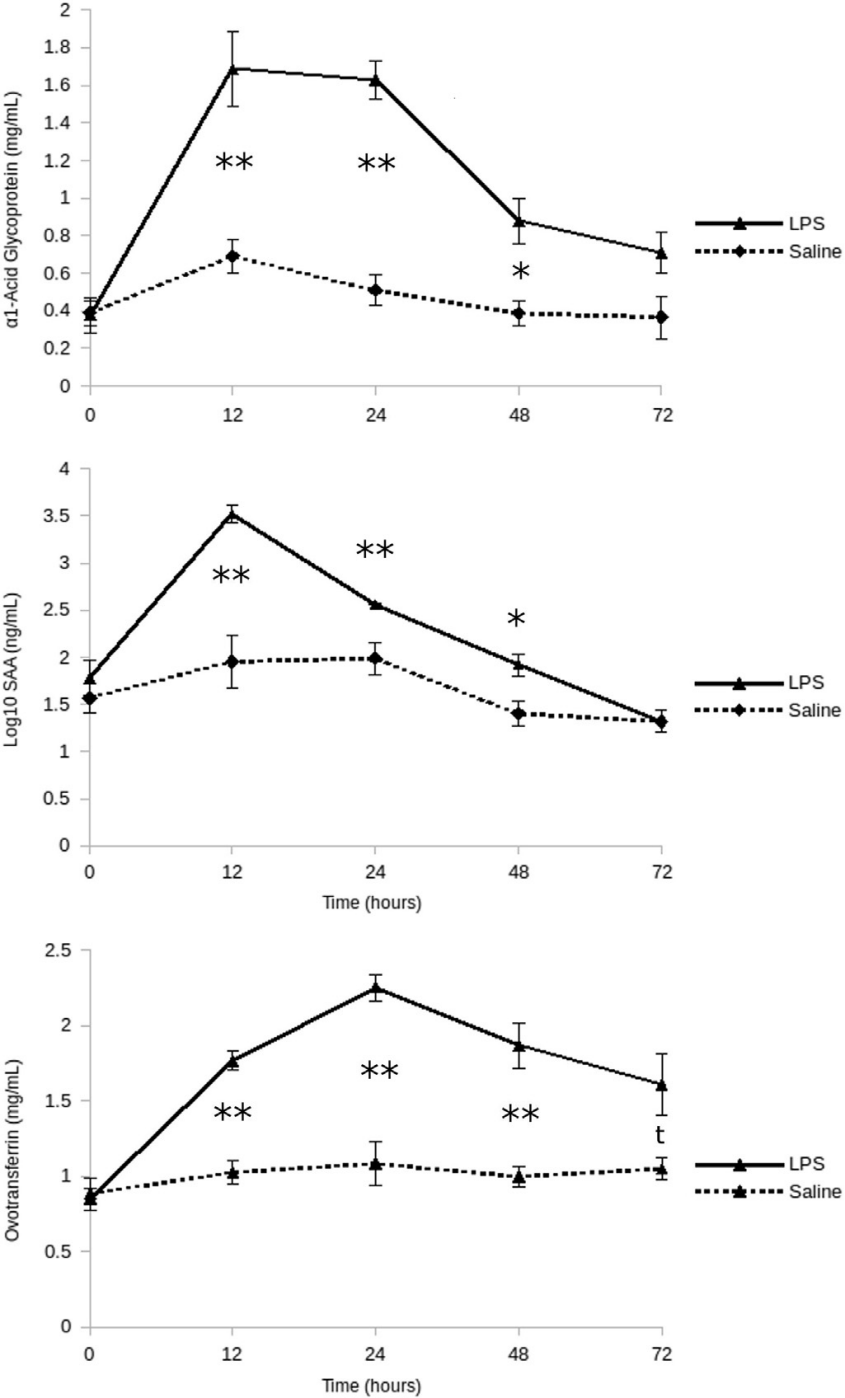
ELISA quantification of alpha-1-acid glycoprotein (AGP), serum amyloid A (SAA), and ovotransferrin (OVT) in different time points. Quantity of each proteins have been represented among time points, for the 2 groups: LPS (continuous line) and saline (dash line). To better visualize difference in SAA, quantities have been transformed by the function log10 for the figure. SEM for each groups and time points have been added. Significance of differences between LPS and saline group for each time point separately have been represented. *p<0.05, **p<0.01 and t: p>0.05.

Fig. 5 represent quantification of 3 proteins (α1-acid glycoprotein, SAA, ovotransferrin) performed by ELISA at 5 time points (0, 12, 24, 48 and 72 h).

**Fig. 6:**
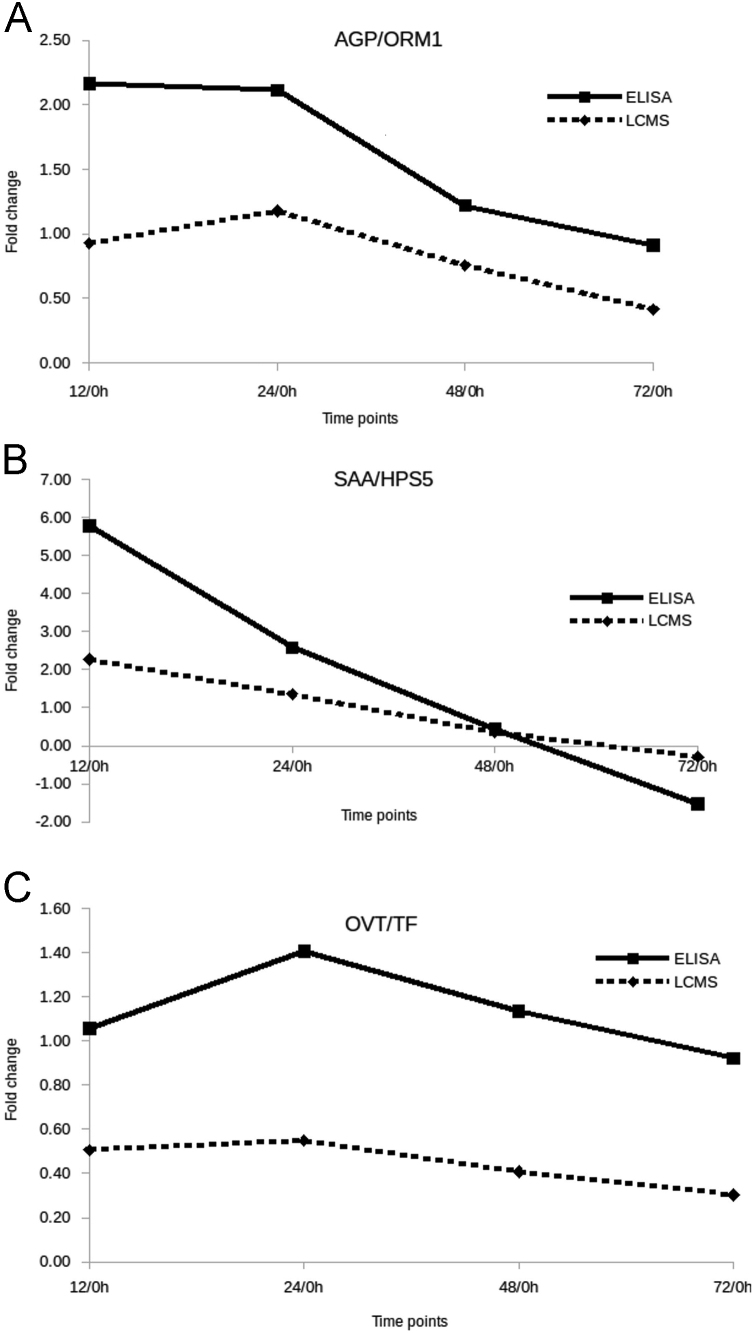
Comparison of 4 fold changes among 5 time points performed by ELISA and LC-MS on alpha-1-acid glycoprotein (AGP), serum amyloid A (SAA), and ovotransferrin (OVT). Fold changes values have been represented for the 3 proteins to establish a comparison between ELISA and LC-MS quantifications: AGP/ORM1 (A), OVT/TF (B), and SAA/HPS5 (C).

Fig. 6 represent differences in fold changes (LPS vs saline) between 4 times points (12, 24, 48, 72 h) and 0 h, to compare ELISA and LS-MS quantification.

Table 1 list proteins significantly different between LPS and saline group, with their associated fold changes and p-values.

**Table 1:** Proteins with significantly differential abundances between LPS and saline groups identified using TMT approach.

**Gene Symbol**			
**(*****Gallus gallus*****)**	**Protein name**	**P-value (FDR)**	**Fold Change**
HPS5	serum amyloid A protein	2.20E-03	1.24
SERPINA3	alpha-1-antiproteinase*	7.89E-07	1.02
HPX	haemopexin*	5.35E-06	0.79
ORM1	alpha 1-acid glycoprotein	1.05E-05	0.66
LCN8	extracellular fatty acid-binding protein precursor	7.62E-03	0.65
TF	ovotransferrin	3.61E-06	0.49
CP	ceruloplasmin*	1.14E-03	0.48
SMC4	condensin complex subunit	1.94E-03	0.47
LOC107051143	complement C3-like*	2.09E-05	0.43
CFD	complement factor D, partial*	1.05E-04	0.34
LOC423629	uncharacterized protein LOC423629*	1.87E-03	0.33
LOC419851	complement regulatory soluble protein	9.47E-03	0.32
PIT54	PIT 54	1.17E-04	0.29
LOC100858647	beta-microseminoprotein-like*	2.99E-03	0.28
FGA	Fibrinogen alpha chain precursor	4.45E-05	0.26
CLU	clusterin isoform X1*	6.86E-03	0.21
FGB	Fibrinogen beta chain precursor	2.46E-04	0.21
FGG	fibrinogen gamma chain precursor	1.10E-04	0.21
C3	complement C3 precursor	1.41E-03	0.19
IGLL1	immunoglobulin light chain variable region, partial	1.79E-02	0.18
CFHR2	complement factor H*	1.64E-05	0.17
SPINK5	ovoinhibitor	2.87E-02	0.17
A2ML4	alpha-2-macroglobulin-like protein 1*	8.86E-03	0.17
VNN1	pantetheinase precursor	3.69E-02	0.17
APOH	beta-2-glycoprotein 1 precursor	3.50E-03	0.15
FETUB	fetuin-B precursor	8.55E-06	0.15
PLG	plasminogen*	3.20E-06	0.12
ATRN	attractin isoform X3*	3.08E-02	0.11
LOC418892	uncharacterized protein	2.78E-02	0.11
CST3	cystatin precursor	2.25E-02	−0.10
TNC	tenascin	4.09E-02	−0.11
ITIH3	inter-alpha-trypsin inhibitor heavy chain H3 isoform X1*	4.21E-02	−0.12
AGRN	basement membrane-specific heparan sulfate proteoglycan core protein precursor	2.51E-02	−0.12
ITIH2	inter-alpha-trypsin inhibitor heavy chain H2 precursor	1.96E-02	−0.12
IGFALS	insulin-like growth factor-binding protein complex acid labile subunit isoform X1*	3.71E-03	−0.13
HSPG2	basement membrane-specific heparan sulfate proteoglycan core protein*	1.79E-02	−0.13
HRG	histidine-rich glycoprotein*	1.51E-02	−0.14
COL5A1	alpha 1 (V) collagen	2.79E-02	−0.14
KNG1	kininogen-1*	1.19E-03	−0.14
C1QTNF3	complement C1q tumor necrosis factor-related protein 3 isoform X1*	8.85E-03	−0.14
F13A1	coagulation factor XIII A chain	4.15E-02	−0.14
LUM	lumican precursor	2.45E-03	−0.14
AHSG	alpha-2-HS-glycoprotein*	3.98E-02	−0.14
GIF	hypothetical protein RCJMB04_7i4	3.80E-02	−0.15
PROS1	vitamin K-dependent protein S*	1.42E-02	−0.15
APOA2	apolipoprotein A-II*	2.47E-02	−0.16
ANPEP	aminopeptidase, partial	2.11E-02	−0.16
LOC107056848	cadherin-5-like, partial*	7.56E-03	-0.17
VTN	vitronectin precursor	1.59E-05	−0.17
CL2	ribonuclease CL2 precursor	1.69E-02	−0.17
LOC107055759	vitamin K-dependent protein S-like*	7.86E-03	−0.17
APOA1	apolipoprotein A-I	1.64E-05	−0.18
FBLN1	fibulin-1, isoform D precursor	3.85E-02	−0.18
APOA4	apolipoprotein A-IV precursor	4.10E-04	−0.19
IL6ST	interleukin-6 receptor subunit beta precursor	4.32E-05	−0.20
TFRC	chicken transferrin receptor	2.19E-04	−0.20
SPARC	basement-membrane protein 40 precursor	3.29E-02	−0.20
F13B	coagulation factor XIII B chain isoform X1*	9.61E-03	−0.20
LOC100857892	sushi, nidogen and EGF-like domain-containing protein 1 isoform X1*	8.56E-03	−0.20
LOC107050076	IgGFc-binding protein-like, partial*	4.78E-03	−0.21
SERPINF1	pigment epithelium-derived factor precursor	3.03E-06	−0.21
CLEC3B	tetranectin precursor	7.32E-03	−0.22
SERPINC1	antithrombin-III*	1.39E-06	−0.23
SERPINA4	serpin peptidase inhibitor, clade A (alpha-1 antiproteinase, antitrypsin), member 4 precursor	3.23E-04	−0.23
LOC771012	coagulation factor X-like*	4.19E-03	−0.23
ENO1	alpha-enolase	6.46E-03	−0.23
LOC100858068	IgGFc-binding protein-like, partial*	2.32E-02	−0.23
RBP4A	E Chain E, Retinol Binding Protein Complexed With Transthyretin	2.26E-04	-0.25
FN1	fibronectin, partial	9.44E-03	−0.26
SERPINA5	alpha-1-antitrypsin isoform X1*	2.49E-04	−0.28
TGFBI	transforming growth factor-beta-induced protein ig-h3 precursor	3.84E-06	−0.28
HBG2	beta-globin	1.46E-02	−0.29
LCAT	lecithin-cholesterol acyltransferase, partial	1.14E-02	−0.29
C4	complement C4 precursor	3.37E-02	−0.30
ALPP	intestinal-type alkaline phosphatase*	1.27E-02	−0.30
POSTN	periostin precursor	3.16E-06	−0.32
COL1A1	collagen alpha-1(I) chain*	2.52E-05	−0.36
SCARA5	scavenger receptor class A member 5 isoform X1*	1.97E-02	−0.36
LOC776376	pentraxin-related precursor	1.82E-02	−0.39
HBAD	alpha-D globin	3.10E-02	−0.43
COL6A1	collagen alpha-1(VI) chain precursor	2.07E-06	−0.44
CPN2	carboxypeptidase N subunit 2*	2.11E-05	−0.46
LRRC15	uncharacterized protein LRRC15*	2.11E-05	−0.46
HBAA	hemoglobin subunit alpha-A chain	7.37E-03	−0.48
KRT8	keratin, type II cytoskeletal 8*	3.35E-02	−0.50
LOC107055417	keratin, type II cytoskeletal 8-like, partial*	3.35E-02	−0.50
COL1A2	collagen alpha-2(I) chain precursor	7.52E-04	−0.51

All proteins belong to the *Gallus gallus* proteome (UniprotKB).

* Proteins predicted in Gallus gallus, with no evidence of existence to date at protein, transcript or homology levels.

Table 2 list proteins significantly different between LPS and saline group which are affected by time effect, with associated fold changes among time and p-values.

**Table 2:** Proteins with significantly differential abundances during time in chicken challenged with *Escherichia coli* lipopolysaccharide endotoxin.

**Gene symbol**	**P value (FDR)**	**Fold change (12 h/0 h)**	**Fold change (24 h/0 h)**	**Fold change (48 h/0 h)**	**Fold change (72 h/0 h)**
A2ML4	1.57E-02	0.34	0.44	0.31	0.19
AHSG	1.06E-02	−0.43	−0.15	−0.04	0.13
APOA2	1.37E-02	−0.36	−0.49	−0.19	−0.30
C3	4.39E-02	0.18	0.35	0.35	0.19
CFD	1.89E-02	0.42	0.61	0.62	0.43
COL1A1	3.39E-02	−0.62	−0.42	−0.42	−0.23
CP	3.16E-03	1.02	1.05	0.51	0.21
FGA	9.93E-03	0.29	0.38	0.23	0.03
FGB	1.03E-02	0.26	0.34	0.24	0.03
FGG	9.19E-03	0.23	0.33	0.23	0.01
HPS5	5.44E-03	2.27	1.35	0.35	-0.29
HPX	7.81E-03	0.76	1.20	1.21	0.94
ITIH2	4.46E-02	−0.19	−0.05	0.02	0.04
LCN8	1.82E-02	1.78	1.02	0.66	0.21
ORM1	5.01E-03	0.93	1.18	0.76	0.42
POSTN	2.18E-02	0.06	−0.30	−0.35	−0.15
SERPINA3	4.31E-03	1.24	1.35	0.99	0.73
TF	1.54E-02	0.51	0.55	0.41	0.30
TGFBI	1.50E-02	−0.34	−0.52	−0.29	−0.24

Table 3 list GO terms associated by LPS challenge, with their associated p-values.

**Table 3:** GO terms over-represented in chicken challenged with *Escherichia coli* lipopolysaccharide endotoxin *vs* saline group.

**GOID**	**GOTerm**	**Term p-value**	**Genes**	**Cluster**	**Redundant**	**Leader**	**Group**
GO:0072378	blood coagulation, fibrin clot formation	1.18E-12	6	No specific			GO:0052547
**GO:0031589**	**cell-substrate adhesion**	**1.76E-04**	**7**	**No specific**		**Yes**	**GO:0031589**
GO:0015893	drug transport	6.75E-03	4	Negative			GO:1902042
GO:0035987	endodermal cell differentiation	5.95E-04	3	Negative			GO:0052547
GO:0030198	extracellular matrix organization	6.46E-03	4	Negative			GO:0043062
**GO:0043062**	**extracellular structure organization**	**8.25E-04**	**6**	**Negative**		**Yes**	**GO:0043062**
GO:0030195	negative regulation of blood coagulation	1.75E-06	5	Positive	Yes		NA
GO:0010951	negative regulation of endopeptidase activity	9.55E-18	16	Negative	Yes		NA
GO:2000352	negative regulation of endothelial cell apoptotic process	4.89E-04	3	Positive			GO:1902042
**GO:1902042**	**negative regulation of extrinsic apoptotic signaling pathway via death domain receptors**	**4.17E-04**	**3**	**Positive**		**Yes**	**GO:1902042**
GO:0031639	plasminogen activation	3.14E-04	3	Positive			GO:0052547
GO:0070527	platelet aggregation	4.19E-03	3	Positive	Yes		NA
GO:0022409	positive regulation of cell-cell adhesion	3.36E-03	4	Positive			GO:0031589
GO:0045921	positive regulation of exocytosis	4.19E-03	3	Positive	Yes		NA
GO:0090277	positive regulation of peptide hormone secretion	4.94E-04	4	Positive			GO:1902042
GO:0050714	positive regulation of protein secretion	4.76E-03	4	Positive	Yes		NA
GO:0045907	positive regulation of vasoconstriction	6.99E-04	3	Positive			GO:0052547
GO:0072376	protein activation cascade	2.79E-13	9	No specific			GO:0052547
GO:0016485	protein processing	1.58E-03	5	Positive	Yes		NA
GO:0030193	regulation of blood coagulation	6.70E-08	7	No specific	Yes		NA
GO:0010810	regulation of cell-substrate adhesion	9.15E-03	3	Negative	Yes		NA
GO:0051336	regulation of hydrolase activity	1.52E-10	20	Negative			GO:0052547
**GO:0052547**	**regulation of peptidase activity**	**1.62E-17**	**18**	**Negative**		**Yes**	**GO:0052547**
GO:0051592	response to calcium ion	4.15E-03	3	Positive			GO:1902042
GO:0042060	wound healing	7.79E-07	10	Negative	Yes		NA

GO terms were determined by Cytoscape/ClueGO and then analysed by REVIGO. GO terms in bold have been identified as the most representative of their GO group by the tool REVIGO.

Table 4 list GO terms associated by LPS challenge and time, with their associated p-values.

**Table 4:** GO terms over-represented in chicken challenged with *Escherichia coli* lipopolysaccharide endotoxin during time.

**GOID**	**GOTerm**	**Term p-value**	**Genes**	**Redundant**	**Leader**	**Group**
GO:0006953	acute-phase response	7.12E-06	3			GO:0072376
GO:0010951	negative regulation of endopeptidase activity	3.40E-05	4			GO:0072376
**GO:0072376**	**protein activation cascade**	**6.11E-07**	**4**		**Yes**	**GO:0072376**
**GO:0034116**	**positive regulation of heterotypic cell-cell adhesion**	**4.60E-07**	**3**		**Yes**	**GO:0034116**
GO:0051592	response to calcium ion	5.96E-05	3			GO:0072376
GO:0070527	platelet aggregation	5.32E-05	3	Yes		GO:0072376
GO:0045921	positive regulation of exocytosis	5.32E-05	3	Yes		GO:0072376
GO:0042730	fibrinolysis	3.15E-06	3			GO:0072376
GO:0045907	positive regulation of vasoconstriction	7.12E-06	3			GO:0072376
GO:0050714	positive regulation of protein secretion	1.72E-04	3	Yes		GO:0072376
GO:0090277	positive regulation of peptide hormone secretion	4.96E-05	3			GO:0072376
GO:0031639	plasminogen activation	3.15E-06	3			GO:0072376
GO:1902042	negative regulation of extrinsic apoptotic signaling pathway via death domain receptors	3.99E-06	3			GO:0072376
GO:2000352	negative regulation of endothelial cell apoptotic process	4.80E-06	3			GO:0072376

GO terms were determined by Cytoscape/ClueGO and then analysed by REVIGO. GO terms in bold have been identified as the most representative of their GO group by the tool REVIGO.

Table 5 present different group and time effects for the proteins SAA, AGP and OVT, quantified by ELISA.

**Table 5:** P-values of group and time effects on alpha-1-acid glycoprotein (AGP), serum amyloid A (SAA), and ovotransferrin (OVT) proteins quantified by ELISA.

**Protein**	**Group**	**Group x time**	**Time - LPS group**	**Time – saline group**
AGP	<0.001	<0.001	<0.001	NS
SAA	<0.001	<0.001	<0.001	NS
OVT	<0.001	<0.001	<0.001	NS

NS: Not Significant (p>0.05). Group effect was assessed between LPS and saline samples by Wilcoxon-test (2-sided). Mixed Group x Time and Time effects were assessed by a Kruskal-Wallis test.

Table 6 present results about time effect on proteins AGP, SAA and OVT, between LPS stimulated samples and controls, and inside the LPS-stimulated group.

**Table 6:** Time effect for alpha-1-acid glycoprotein (AGP), serum amyloid A (SAA), and ovotransferrin (OVT) proteins quantified by ELISA. A. Effect of group (LPS *versus* saline) for each time point, saline used as reference to compare. B. Effect of time on proteins fold change compared with 0 h (reference level).

**A. Group effect (LPS versus saline) for each time point**						
		**0 h**	**12 h**	**24 h**	**48 h**	**72 h**
**AGP**	Fold change	−0.04	1.29	1.68	1.18	0.96
	P value	NS	<0.01	<0.01	<0.05	NS
**SAA**	Fold change	0.73	5.21	1.90	1.71	0.01
	P value	NS	<0.01	<0.01	<0.05	NS
**OVT**	Fold change	−0.06	0.78	1.05	0.90	0.62
	P value	NS	<0.01	<0.01	<0.01	<0.1

**B. Time effect in LPS group compared with 0 h**						
		**12 h/0 h**	**24 h/0 h**	**48 h/0 h**	**72 h/0 h**	
**AGP**	Fold change	2.16	2.11	1.22	0.91	
	P value	<0.01	<0.01	<0.05	NS	
**SAA**	Fold change	5.77	2.58	0.44	−1.53	
	P value	<0.01	<0.01	NS	NS	
**OVT**	Fold change	1.06	1.41	1.13	0.92	
	P value	<0.01	<0.01	<0.01	<0.01	

NS: Not Significant (p>0.05). Differences were assessed with a Wilcoxon-test (2-sided).

## 2 Experimental design, materials and methods

In March and April 2017 one day old, Ross 308 broiler chicks (PD Hook Hatcheries Ltd, Bampton, UK), were fitted with unique wing tags and housed in 4 groups of 14 in adjacent 1 m × 2 m pens on a litter of wood shavings on the University of Glasgow Cochno Farm & Research Centre. Broiler mash and water were available *ad libitum*. From the second day, one group per day was handled and moved into the trial room. All chickens were confirmed to be climatized to handling by 15 days old. Room temperature was maintained within the thermal neutral zone at 18 °C (range 18.0–18.3) and a 20 h:4 h light: dark cycle was implemented.

The experiment commenced when the chickens were 15 days old. Twenty four birds were injected subcutaneously (SC) at time point 0, with *Escherichia coli* lipopolysaccharide (LPS from E. coli O111:B4 purified by phenol extraction, L2630-25MG; Sigma-Aldrich, Dorset, UK) (2 mg/kg body weight) in a volume of 0.5 mL as the treatment group and another 24 birds injected SC by sterile normal saline (0.5 mL) as a control group. There were 5 blood sampling time points; pre (0 h) and post injection (PI) at 12, 24, 48, and 72 h. Plasma was collected from the same 6 chicken in the treated group and from the same 6 chicken in the untreated group, subsequently, at each time point for further analyses by proteomic and immunoassay methods. The remaining 18 birds in each group were not used in the plasma proteome investigation. Approximately 1.2 mL of blood was collected from the wing vein using heparinized tubes at each time point. The heparinised blood was centrifuged (3000g) for 15 min at 4 °C and the plasma aspirated and immediately frozen at −20 °C.

After the trial, all chickens were culled by over dose (1.5–2 mL/bird) *i.v.* injection of barbiturate (Euthatal 200 mg/mL, Merial, Woking, UK). Research was conducted under Home Office license (60/4466), and approved by ethical review of the University of Glasgow, MVLS College Ethics Committee.

## 3 Proteomic investigation of chicken plasma

Proteomic analysis of chicken plasma samples was performed by applying TMT-based quantitative gel-free approach as described previously [2]. In brief, after total protein concentration determination using BCA assay (Thermo Scientific, Rockford, USA), 35 µg of total plasma proteins from samples and internal standard (pool of all samples) were diluted to a volume of 50 µL using 0.1 M triethyl ammonium bicarbonate (TEAB, Thermo Scientific, Rockford, USA), reduced by adding 2.5 µL of 200 mM DTT (60 min, 55 °C) (Sigma Aldrich, St. Louis, MO, USA), alkylated by adding 2.5 µL of 375 mM IAA (30 min, room temperature in the dark) (Sigma Aldrich, St. Lois, MO, USA) and acetone-precipitated (addition of 300 µL, overnight, −20 °C). Protein pellets were collected subsequently by centrifugation (8000*g*, 4 °C), dissolved in 50 µL of 0.1 M TEAB and digested using 1 µL of trypsin (1 mg/mL, Promega; trypsin-to-protein ratio 1:35, at 37 °C overnight). TMT sixplex reagents (Thermo Scientific, Rockford, IL, USA) were prepared according manufacturer׳s procedure and an amount of 19 µL of the appropriate TMT label was added to each sample used for the labelling reaction (60 min, room temperature) which was quenched using 5% hydroxylamine (Sigma-Aldrich, St. Louis, MO, USA). Five TMT-modified peptide samples were combined with the internal standard (labelled with TMT *m*/*z* 126) into the new tube, aliquoted, dried and stored at −20 °C for further analysis. A total of 30 samples (6 chicken at 5 time points) from treated and 30 samples from control chicken led to 12 individual TMT experiments with the inclusion of internal standards in each experiment.

High resolution LC-MS/MS analysis of TMT-labelled peptides was carried out using an Ultimate 3000 RSLCnano system (Dionex, Germering, Germany) coupled to a Q Exactive Plus mass spectrometer (Thermo Fisher Scientific, Bremen, Germany). Peptides were loaded onto the trap column (C18 PepMap100, 5 µm, 100 A, 300 µm × 5 mm), desalted for 12 min at the flow rate of 15 uL/min and separated on the analytical column (PepMap™ RSLC C18, 50 cm × 75 μm) using linear gradient 5–45% mobile phase B (0.1% formic acid in 80% ACN) over 120 min, 45% to 90% for 2 min, held at 80% for 2 min and re-equilibrated at 5% B for 20 min at the flow rate of 300 nL/min. Loading solvent consisted of 0.1% formic acid and 2% ACN in water, while mobile phase A contained 0.1% formic acid in water. Ionisation was achieved using nanospray Flex ion source (Thermo Fisher Scientific, Bremen, Germany) containing a 10 μm-inner diameter SilicaTip emitter (New Objective, USA). The MS operated in positive ion mode using DDA Top8 method. The lock mass feature was not in use in this experiment. Full scan MS spectra were acquired in range from *m*/*z* 350.0 to *m*/*z* 1800.0 with a resolution of 70,000, 120 ms injection time, AGC target 1E6, a±2.0 Da isolation window and the dynamic exclusion 30 s. HCD fragmentation was performed at step collision energy (29% and 35% NCE) with a resolution of 17,500 and AGC target of 2E5. Precursor ions with unassigned charge state, as well as charge states of +1 and more than +7 were excluded from fragmentation. MS2 was operated in centroid mode.

For peptide identification and relative quantification the SEQUEST algorithm implemented into Proteome Discoverer (version 2.0., Thermo Fisher Scientific) was used. Database search against *Gallus gallus* FASTA files downloaded from NCBI database (7/12/2017, 46105 entries, NCBI *Gallus gallus* Annotation Release ID 103) was performed according to the following parameters: two trypsin missed cleavage sites, precursor and fragment mass tolerances of 10 ppm and 0.02 Da, respectively; carbamidomethyl (C) fixed peptide modification, oxidation (M), deamidation (N,Q) and TMT sixplex (K, peptide N-terminus) dynamic modifications. The false discovery rate (FDR) for peptide identification was calculated using the Percolator algorithm in the Proteome Discoverer workflow based on the search results against a decoy database and was set at 1% FDR. Only proteins with at least two unique peptides and less than 5% FDR were reported as reliable identification. Protein quantification was accomplished by correlating the relative intensities of reporter ions extracted from tandem mass spectra to that of the peptides selected for MS/MS fragmentation The internal standard was used to compare relative quantification results for each protein between the experiments (sixplexes). Peptide and protein identification data are shown in Supplementary file 1.

The mass spectrometry proteomics data have been deposited to the ProteomeXchange Consortium via the PRIDE partner repository [3] with the dataset identifier PXD009399.

## 4 Statistical and bioinformatics analysis of the chicken plasma proteome

### 4.1 Statistics for proteomics

All statistics were performed using R (v3.4.3) [4] under RStudio environment (v1.0.143) [5]. Infection effect (saline versus LPS) and time effect in infection groups (0 h,12 h,24 h,48 h,72 h in saline and LPS separately) were considered for investigation. A peptide was not considered for the analysis if one of its group (infection, time or infection x time) had more of 50% of missing data after LC-MS identification and quantification. Filtered data are shown in Supplementary file 2.

A two-way ANOVA was performed to model the effect of treatment and time on the quantity of the peptides, using a linear regression model. Distribution of residuals generated by the ANOVA was accessed by a Shapiro-Wilk test. A Kruskal-Wallis test was performed to access the effect of treatment and time on peptides quantity using the R package “PMCMRplus” [6]. Due to multiple comparisons performed, a local False Discovery Rate was applied using the R package “qvalue” [7]. Each p-value was transformed by the function -log10(x). Obtained data are presented in Fig. 1, Fig. 2, as well as in Table 1, Table 2 of Ref [1].

Fold change between the 2 groups has been calculated by the function log2(Mean(Group2)/Mean(Group1)). A volcano plot was designed using the R package “plotly” [8]. Plots were generated with the “ggplot2” package [9]. Spearman׳s correlation were calculated to estimate the relationship between ELISA and LC-MS quantifications for the proteins AGP, SAA and OVT (Fig. 6 of Ref [1]).

All operations were scripted in R to assure the automatization of the statistics pipeline to all peptides.

### 4.2 Bioinformatics

Proteins ID (*Gallus gallus*) were converted into Gene ID (*Gallus gallus*) by the platform DAVID (david-d.ncifcrf.gov/conversion.jsp) conversion tool. Gene Ontology enrichment analysis was performed using the Cytoscape (v3.6.0) [10] plugin ClueGO (v2.5.0) [11] on GO-Biological Processes (08/03/2018).

For treatment effect (LPS versus saline), two clusters of proteins differentially expressed between the 2 groups were set: one cluster for over-expressed proteins following LPS treatment, the other for proteins exhibiting lower-expression following LPS. The analysis was performed using the following parameters: evidence code=All, GO levels 3 to 15, minimal number of gene=3, minimal percentage of gene=3, Kappa score threshold=0.4, p-values corrected by Bonferroni step down.

For time effect, differentially expressed proteins with time were analyzed at once using the following parameters: evidence code=All, GO levels 3 to 8, minimal number of gene=3, minimal percentage of gene=3, Kappa score threshold=0.4, p-values corrected by Bonferroni step down.

The two lists of GO terms over-expressed in the context of group and time effects were submitted to an analysis by REVIGO (revigo.irb.hr) [12] to remove redundant GO terms and group similar terms based on their description. For both analyses, the database used was *Gallus gallus*, with the SimReal semantic similarity measure.

Pathways of relationship between GO terms filtered according to REVIGO with their proteins/genes were designed on Cytoscape. Fold change data was included for the time effect analysis on samples from the LPS treated group. Pathway analysis data are shown in Fig. 3, Fig. 4, as well as in Tables 3 and 4 of Ref [1].

## 5 Measurement of acute phase protein concentrations

### 5.1 Immunoassays

The concentrations of AGP, SAA and OVT were determined in the plasma according to previously described procedures [13]. The ELISA assays for chicken AGP and SAA were obtained from Life Diagnostics Inc (West Chester, USA). They were performed according to the manufacturer׳s instructions with a dilution factor for the plasma samples of 1:10,000 for AGP and 1:20 for SAA. Each individual sample was run in duplicate. The plasma concentration of OVT was assessed by radial immunodiffusion (RID) using specific antibody for chickens OVT as described previously [50]. Data are presented in Fig. 5 of Ref [1].

### 5.2 Statistics for immunoassays

Statistics on immunoassay were performed by non-parametric tests due to group size and distribution. Group effect was assessed by a Wilcoxon-test (2-sided), and a Kruskal-Wallis test was used to assess mixed effect Group x Time on all groups and Time effect on LPS and saline groups separately. For each time point (0 h/12 h/24 h/48 h/72 h), difference between LPS and saline was assessed by a Wilcoxon-test (2-sided) and fold change of expression calculated between times 12 h/24 h/48 h/72 h versus 0 h in LPS group. Correlation between these proteins was assessed on LPS group by a Spearman rank test. Immunoassays-related statistical data are shown in Tables 5 and 6 of Ref. [1].
