# Supplementary material for: Integrated dataset on acute phase protein response in chicken challenged with Escherichia coli lipopolysaccharide endotoxin
Source: Data Brief. 2018 Oct 17;21:684–99. doi: 10.1016/j.dib.2018.09.103 (PMC6205363; doi:10.1016/j.dib.2018.09.103)
Supplement: Supplementary file 1 — Supplementary material. [file mmc1.doc]

**Conflict of interest statement**

Authors have nothing to disclose.
